# Position-Specific Match Demands in Professional Soccer Assessed Using GPS Technology: A Repeated-Measures Analysis

**DOI:** 10.3390/sports14090391

**Published:** 2026-09-04

**Authors:** Efstathios Papadopoulos, Lazaros Vardakis, Maria Papadopoulou, Konstantinos Papadopoulos, Stefanos Katsikas, Glykeria Tsentidou

**Affiliations:** 1 Laboratory for the Evaluation of Human Biological Performance, School of Physical Education and Sport Science, Aristotle University of Thessaloniki, 57001 Thessaloniki, Greece; epapadoab@phed.auth.gr (E.P.); vardakis@live.com (L.V.);; 2 Department of Sports Coaching and Physical Education, School of Sports Science and Physical Education, Metropolitan College, 46 Egnatia Str, 54625 Thessaloniki, Greece; 3 School of Medicine, Faculty of Health Sciences, Aristotle University of Thessaloniki, 54124 Thessaloniki, Greece; papadopouloum565@gmail.com; 4 Department of Physics, School of Science, Democritus University of Thrace, Kavala Campus, 65404 Kavala, Greece; 5 Department of Psychology, School of Psychology, Metropolitan College, 46 Egnatia Str, 54625 Thessaloniki, Greece; 6 Department of Speech and Language Therapy, School of Health Sciences, Metropolitan College, 46 Egnatia Str, 54625 Thessaloniki, Greece

**Keywords:** soccer, global positioning system, external load, high-speed running, sprinting, accelerations, decelerations, mixed-effects model

## Abstract

Playing position influences soccer match demands, but comparisons may be biased by unequal playing time and repeated observations from the same players. This retrospective observational study compared GPS-derived running and change-of-velocity demands across five positional groups while accounting for within-player clustering and match exposure. The primary analysis included 84 player-match observations from 21 male professional outfield players across six competitive matches. Linear mixed-effects models included playing position and match as fixed effects, log-transformed playing duration as a covariate, and player as a random intercept. After Holm correction, position was associated with total distance, high-intensity running distance at ≥19 km/h, distance at ≥25 km/h, sprint count, peak speed, accelerations at ≥3 m/s^2^, and decelerations at ≤−3 m/s^2^ (adjusted *p* ≤ 0.020). At equivalent exposure, central defenders recorded approximately 49% less high-intensity running than wingers and fewer sprints, accelerations, and decelerations than several other groups. Midfielders achieved a 3.06 km/h lower adjusted peak speed than side defenders/full-backs. Findings were robust to two sensitivity analyses. Position-specific monitoring should combine accumulated match dose with exposure-adjusted estimates and account for repeated observations when players contribute data from multiple matches.

## 1. Introduction

Soccer match performance is characterized by prolonged low-intensity activity interspersed with high-speed running, sprinting, accelerations, decelerations, and rapid changes in direction. Total distance is therefore an incomplete representation of competitive demand because similar movement volumes may be accumulated through markedly different intensity and mechanical profiles [1,2,3]. Contemporary wearable tracking systems permit these components to be quantified during match play and have become central to training prescription, recovery planning, and return-to-performance decision-making [4,5].

Playing position is a consistent source of variation in match-running demand. Side defenders/full-backs and wingers frequently perform overlapping runs, defensive recovery actions, and high-speed transitions, whereas central defenders generally accumulate less distance at very high velocities. Midfielders may cover substantial total distance while operating within different acceleration and sprint profiles, and strikers often combine lower exposure with brief high-intensity actions. Evidence from senior, international, and academy football reinforces the need to move beyond team averages when interpreting competitive load [6,7,8,9,10,11,12,13,14,15,16,17].

Unequal playing time and repeated observations from the same players can influence positional comparisons. Raw match totals are directly affected by exposure duration, while repeated player-match records are not statistically independent. These factors should therefore be considered when interpreting position-specific match demands [10,18,19,20].

Speed thresholds also require caution. Absolute thresholds facilitate standardized within-system reporting, yet the same speed can represent a different proportion of each player’s maximal capacity. Distance at ≥25 km/h and sprint counts may consequently reflect both tactical opportunity and individual speed characteristics [5,21,22]. Even so, absolute thresholds remain operationally useful when their limitations are stated and devices and processing procedures are consistent.

The purpose of this study was to compare GPS-derived running and change-of-velocity demands across five playing-position groups while accounting for repeated observations, match effects, and playing duration. It was hypothesized that side defenders/full-backs and wingers would demonstrate greater high-speed running and sprint-related demands than central defenders and midfielders. Accelerations and decelerations were also expected to show pronounced positional differences because their frequency and magnitude are strongly influenced by position-specific tactical actions, including pressing, transitions, defensive recovery, and repeated changes in movement direction [11,12,14].

## 2. Materials and Methods

### 2.1. Study Design and Participants

This retrospective observational study used routine match-monitoring data from 21 male professional outfield soccer players from the first team of a Greek second-tier club. Their mean age was 27.0 ± 3.7 years, body mass was 77.8 ± 6.5 kg, stature was 179.8 ± 6.2 cm, body mass index was 24.1 ± 1.6 kg/m^2^, and body fat percentage was 7.9 ± 2.5%. Skinfold thickness was measured at seven standard anatomical sites using a calibrated mechanical skinfold caliper. Body density was estimated from the sum of the seven skinfolds and age using the Jackson and Pollock equation for men, and body fat percentage was calculated using the Siri equation (% body fat = 495/body density − 450) [23,24]. The players had 17.0 ± 3.8 years of systematic soccer training experience. Players were eligible if they were male first-team outfield players and contributed at least one valid individual match record during the monitored period. Goalkeepers were excluded because goalkeeper-specific match demands were outside the scope of the study. The primary analysis included 84 player-match observations across six official promotion play-off matches. Position groups were central defenders (CD; 14 observations from 4 players), side defenders/full-backs (SD; 15 observations from 4 players), midfielders (M; 22 observations from 5 players), wingers (W; 22 observations from 5 players), and strikers (ST; 11 observations from 3 players). No a priori sample-size calculation was performed because all eligible individual player-match records from the monitored period were included.

### 2.2. GPS Monitoring and Outcomes

Playing duration was recorded in minutes. The seven prespecified GPS-derived outcomes were total distance (m), high-intensity running distance at ≥19.00 km/h (m), distance at ≥25.00 km/h (m), sprint count at ≥25.00 km/h, peak speed (km/h), accelerations at ≥3.00 m/s^2^, and decelerations at ≤−3.00 m/s^2^. The exported speed zones were 19.00–24.99 km/h and ≥25.00 km/h; high-intensity running distance equaled their sum in every record. These absolute speed thresholds were applied uniformly across all players and therefore may not represent the same relative intensity for each individual [5,21,22].

External-load data were collected using 10 Hz GPS units integrated into the Polar Team Pro system (Polar Electro Oy, Kempele, Finland). Each player wore the sensor in a close-fitting monitoring vest, positioned on the upper back between the scapulae, and consistently used the same unit throughout the monitored period to minimize inter-unit variability. To allow sufficient time for satellite acquisition, the GPS units were activated approximately 15 min before the start of each match. A minimum of eight connected satellites was required before data collection. After each match, the units were returned to their docking station, where the recorded data were uploaded and synchronized with the Polar Team Pro platform. The system recorded player movement and heart-rate data during routine match monitoring. The exported dataset included speed-zone, sprint, acceleration/deceleration, heart-rate, training-load, power-zone, RR-interval, and HRV fields [4,25,26,27,28]. HDOP values were not available in the retrospective dataset and were therefore not used as an additional signal-quality criterion. Detailed diagnostic information on signal loss, implausible speed values, and recording artefacts was not available in the retrospective export; therefore, no additional retrospective exclusion thresholds based on these metrics were applied.

Variables outside the seven prespecified external-load outcomes, including proprietary composite scores (Training Load Score, Muscle Load, and Cardio Load) and perceived-load variables (RPE and sRPE), were not included in the present analysis because their measurement protocols and/or completeness were insufficient for reliable interpretation.

### 2.3. Match Procedures and Data Processing

Individual match records were collected during routine monitoring in six official 2025–2026 Super League 2 promotion play-off matches played between 14 February and 22 March 2026. The source workbook contained six aggregated AVERAGE rows representing summary values rather than individual player-match observations. These rows were excluded before statistical analysis and did not contribute to the analytical dataset. The source workbook contained 85 individual records; one record had no position code and was excluded from the primary analysis, leaving 84 complete observations. Player identity was replaced by anonymized codes for analysis and reporting.

All recorded appearances were retained in the primary analysis, including short substitute appearances, because the models adjusted directly for playing duration. To assess whether brief exposure influenced the conclusions, a sensitivity analysis was restricted to appearances lasting at least 30 min. Position was defined according to the player’s functional match role within the predefined positional categories. The team maintained the same tactical formation across the six monitored matches, with no substantial formation changes recorded during match play. The positional analysis included players who maintained the same functional positional role throughout each match. Changes in laterality within the same positional category (e.g., right to left winger) were not considered a change in position.

The six monitored matches comprised three home and three away fixtures. The six matches were played as follows: Iraklis vs. Asteras Aktor B (3–1; 14 February 2026), Niki Volou vs. Iraklis (0–1; 22 February 2026), Anagennisi Karditsas vs. Iraklis (0–1; 28 February 2026), Asteras Aktor B vs. Iraklis (0–2; 8 March 2026), Iraklis vs. Niki Volou (0–0; 14 March 2026), and Iraklis vs. Anagennisi Karditsas (3–1; 22 March 2026). Across the six promotion play-off matches, the team recorded five wins, one draw, and no defeats and ultimately achieved promotion to the first division. The interval between consecutive matches ranged from 6 to 8 days, allowing a regular recovery period between fixtures. Playing duration included the exposure recorded by the monitoring system; no additional correction for stoppage time was applied. Environmental conditions and surface-specific information were not available in the retrospective export and were therefore not included as covariates.

### 2.4. Statistical Analysis

Descriptive data are presented as medians and interquartile ranges because several outcomes were right-skewed and included zero values. Position-related differences were evaluated with linear mixed-effects models estimated by maximum likelihood (REML disabled) using the Powell optimizer. Each model included playing position and match as fixed effects, centered log-transformed playing duration as a covariate, and a random intercept for player to account for repeated observations. Total distance was log-transformed; high-intensity running distance, distance at ≥25 km/h, sprint count, accelerations, and decelerations were transformed as log(y + 1); peak speed was analyzed on its original scale.

The omnibus position effect was evaluated with a four-degree-of-freedom Wald chi-square test. Pairwise positional contrasts were computed as linear combinations of the fitted fixed effects; two-sided Wald z tests and 95% Wald confidence intervals were derived from the estimated fixed-effect covariance matrix. Holm correction was applied across the seven prespecified outcomes and, following a significant adjusted omnibus test, within the set of pairwise contrasts for that outcome. Effects from log-transformed models are reported as exponentiated ratios with 95% confidence intervals; peak-speed effects are reported as adjusted mean differences. Model convergence, random-intercept variance estimates, and standardized residuals were examined. Statistical significance was set at adjusted *p* < 0.05. Sensitivity analyses repeated the complete modeling procedure after including the single inferred position and after restricting the sample to appearances ≥30 min. Analyses were conducted in Python 3.13.5 using statsmodels 0.14.6.

## 3. Results

### 3.1. Descriptive Match Demands

**Figure 1:**
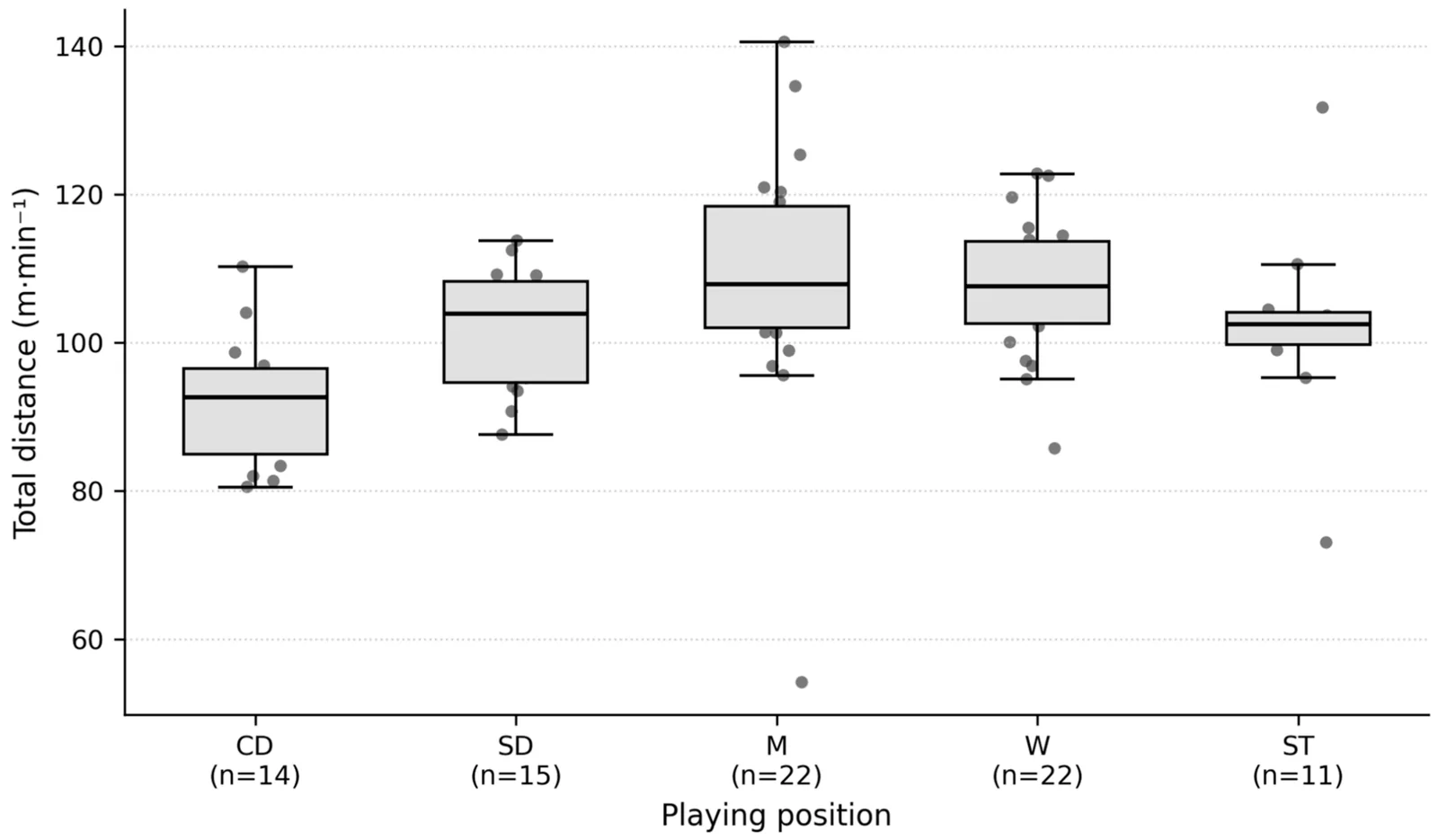
Total distance per minute by playing position. Dots represent player-match observations; boxes show the median and interquartile range, with whiskers extending to 1.5 times the interquartile range. Values are descriptive and unadjusted. CD, Central Defender; M, Midfielder; SD, Side Defender; ST, Striker; W, Winger.

**Figure 2:**
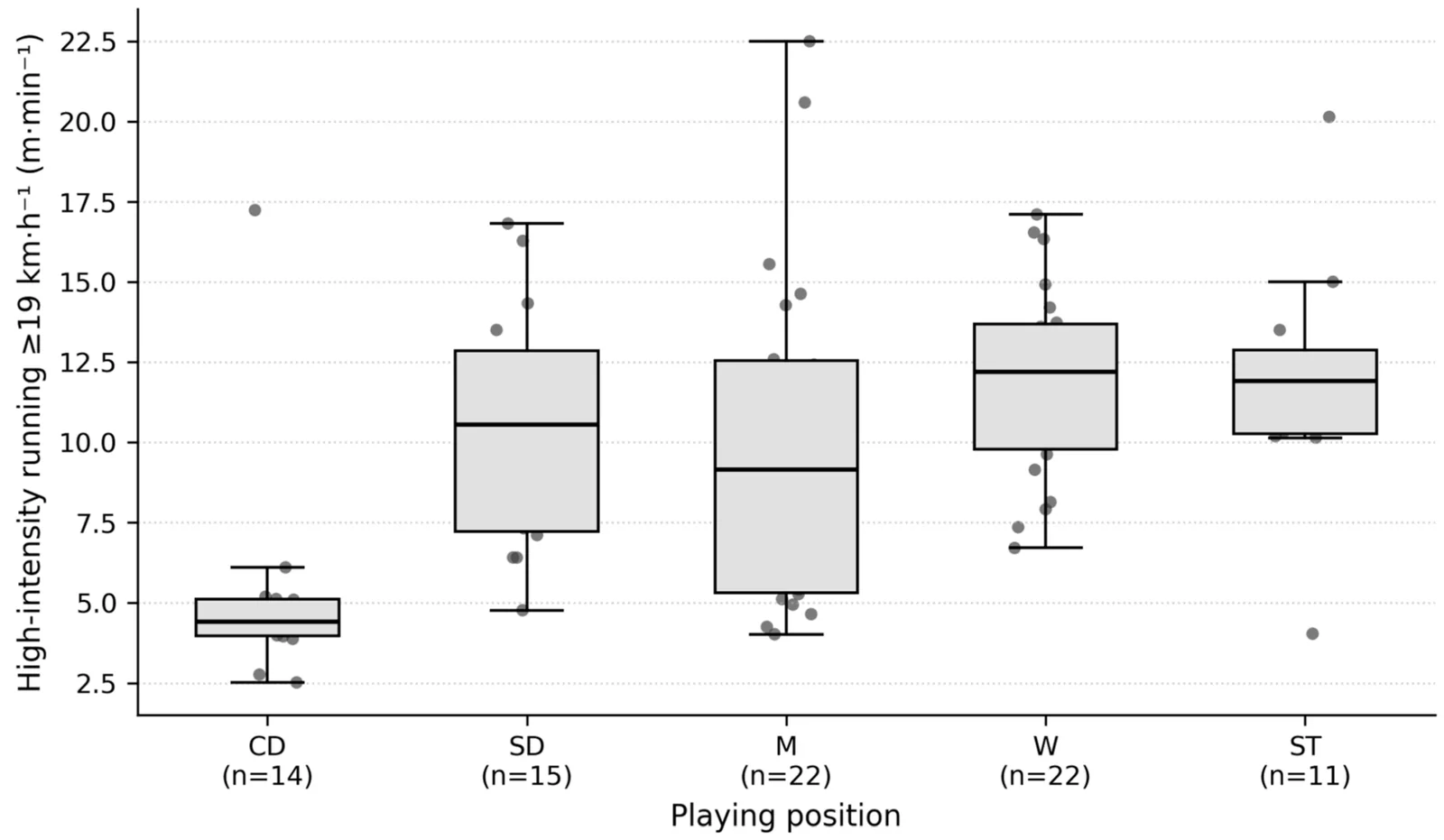
High-intensity running distance at ≥19 km/h per minute by playing position. Dots represent player-match observations; boxes show the median and interquartile range. Values are descriptive and unadjusted.

**Figure 3:**
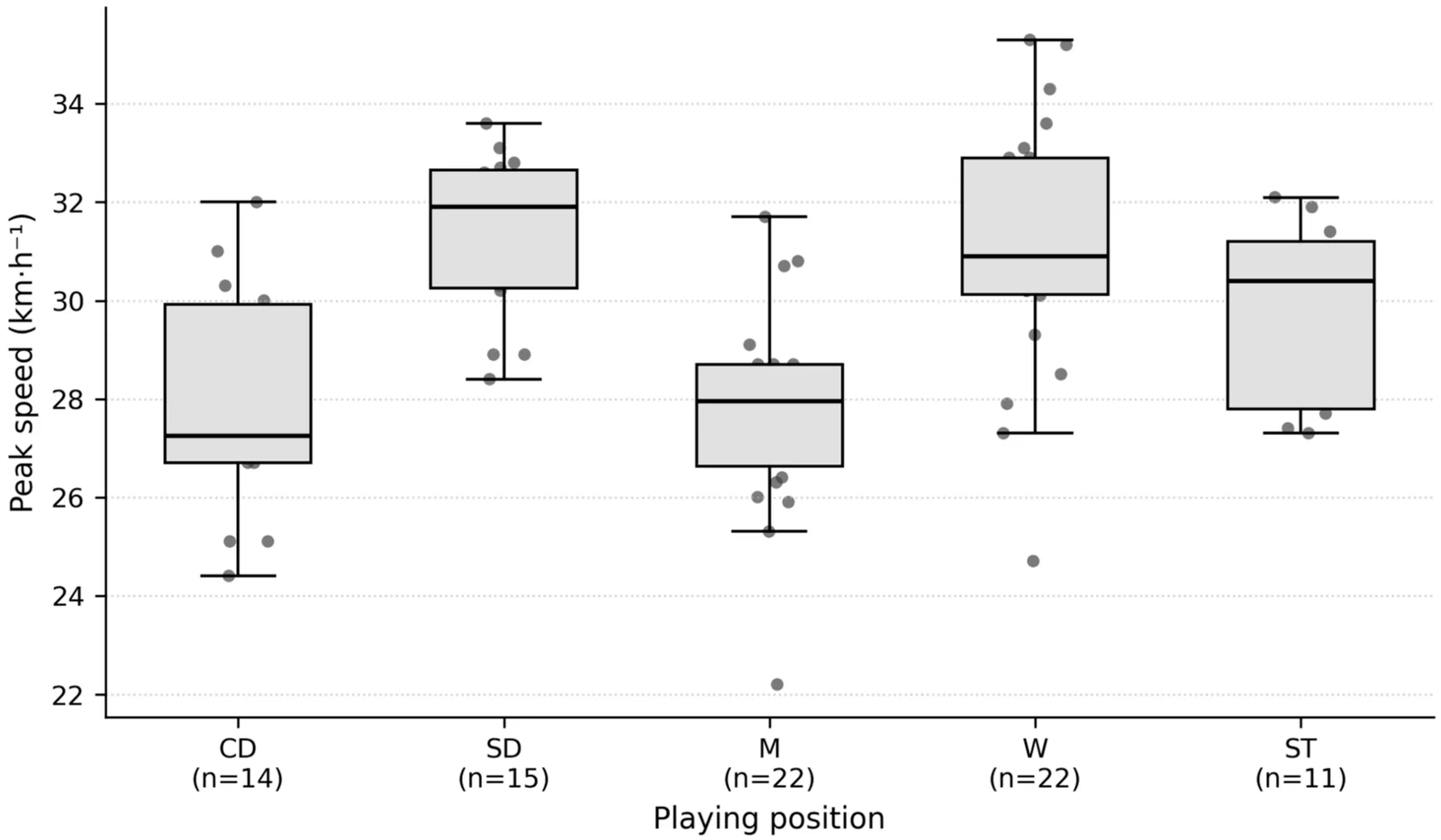
Peak speed by playing position. Dots represent player-match observations; boxes show the median and interquartile range. Values are descriptive and unadjusted; mixed-effects inference is presented in Table 2 and Table 3.

The primary analysis included 84 observations from 21 players. Match exposure varied substantially: CD had a median duration of 95.0 min, whereas ST had a median duration of 52.0 min. In unadjusted descriptive data, SD showed the highest median high-intensity running distance (818 m), distance at ≥25 km/h (242 m), sprint count (13), and peak speed (31.9 km/h). W also displayed high median values for high-intensity running (770 m), sprint count (8.5), accelerations (17), and decelerations (33.5). Table 1 summarizes the unadjusted position-specific distributions, and Figure 1, Figure 2 and Figure 3 provide graphical comparisons of time-normalized running demand and peak speed. Because playing duration differed substantially across positional groups, the unadjusted medians primarily describe the accumulated match dose and should not be interpreted as exposure-equivalent positional differences. Therefore, inferential positional comparisons were based on the exposure-adjusted mixed-effects models presented below.

### 3.2. Mixed-Effects Model Results

After adjustment for match, exposure time, and repeated observations, playing position was associated with all seven prespecified outcomes. All models converged under maximum-likelihood estimation. The Holm-adjusted omnibus p values were 0.002 for total distance, 0.020 for high-intensity running distance, 0.020 for distance at ≥25 km/h, 0.005 for sprint count, 0.013 for peak speed, 0.002 for accelerations, and <0.001 for decelerations. The total-distance model estimated a near-zero player-level random-intercept variance; its position effect remained significant in the ≥30 min sensitivity analysis. Omnibus model results are presented in Table 2.

**Figure 4:**
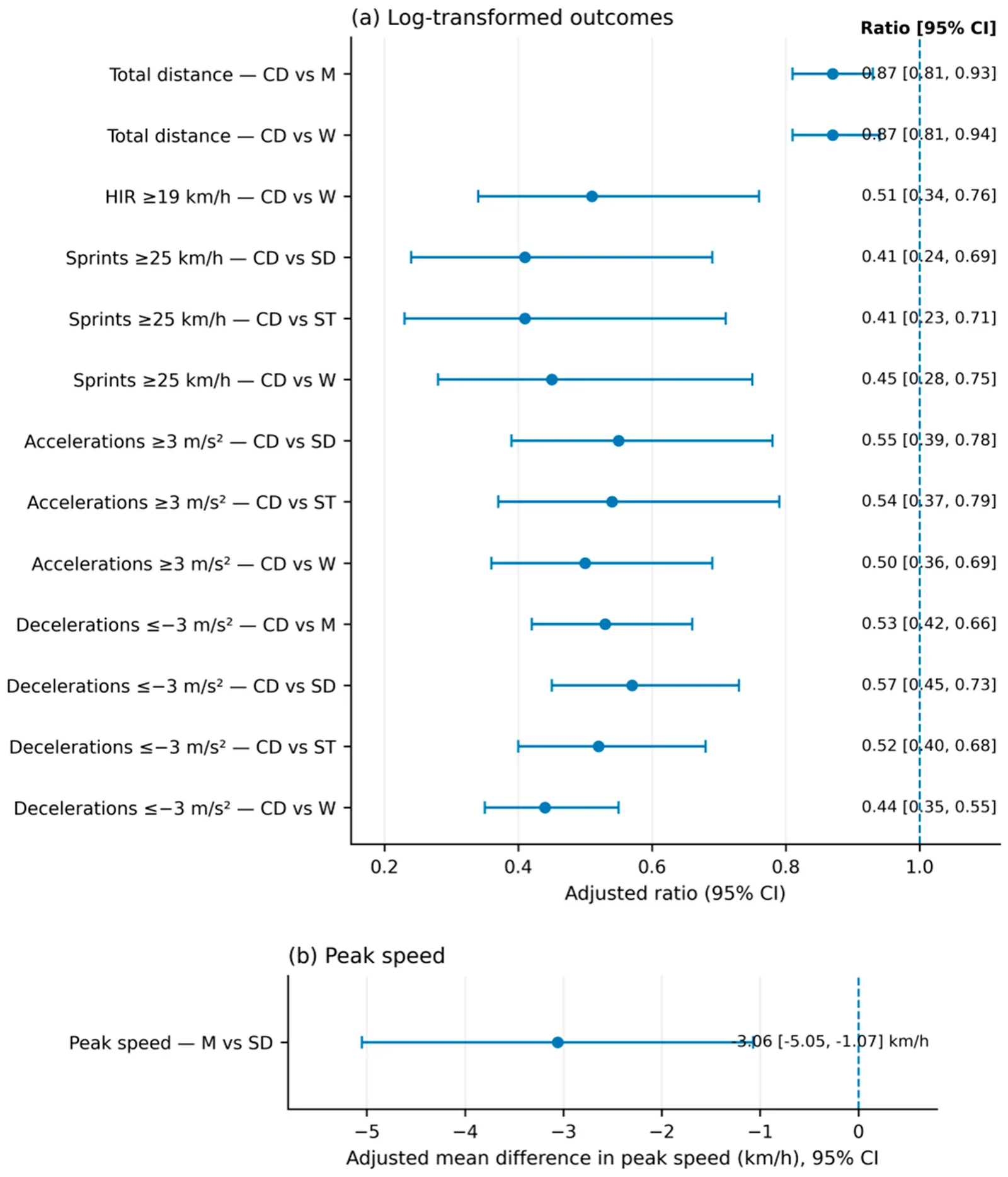
Exposure-adjusted pairwise positional contrasts from the linear mixed-effects models. (**a**) Significant pairwise contrasts for log-transformed outcomes are presented as exponentiated ratios with 95% confidence intervals; ratios below 1.00 indicate lower values in the first-listed positional group. (**b**) The peak-speed contrast is presented as the adjusted mean difference in km/h with its 95% confidence interval. Only pairwise contrasts that remained significant after within-outcome Holm correction are displayed. CD, central defenders; SD, side defenders/full-backs; M, midfielders; W, wingers; ST, strikers; HIR, high-intensity running.

Pairwise contrasts indicated that CD accumulated less adjusted total distance than M (ratio = 0.87, 95% CI [0.81, 0.93]) and W (ratio = 0.87, 95% CI [0.81, 0.94]). CD also recorded approximately 49% less high-intensity running than W (ratio = 0.51, 95% CI [0.34, 0.76]). Although the omnibus test for distance at ≥25 km/h was significant, no individual contrast remained significant after within-outcome Holm correction. For sprint count, CD recorded lower values than SD, ST, and W. CD also performed fewer accelerations than SD, ST, and W and fewer decelerations than every other group. M achieved a 3.06 km/h lower adjusted peak speed than SD (95% CI [−5.05, −1.07] km/h). Significant adjusted pairwise contrasts are detailed in Table 3 and illustrated in Figure 4.

### 3.3. Sensitivity Analyses

The conclusions were unchanged in both sensitivity analyses. When the incomplete record was assigned to W, all seven Holm-adjusted omnibus position effects remained significant (adjusted *p* ≤ 0.027). Restricting the analysis to the 61 appearances lasting at least 30 min also retained significant positional effects for every primary outcome (adjusted *p* < 0.001). 

## 4. Discussion

This study showed that playing position was associated with distinct locomotor and change-of-velocity demands after accounting for repeated player observations, match-to-match variation, and actual playing duration. The clearest differences involved sprint count, peak speed, accelerations, and especially decelerations. Wide roles generally displayed the most demanding high-speed profiles, whereas central defenders performed fewer high-intensity and change-of-velocity actions at equivalent exposure. These patterns are consistent with position-specific findings reported across professional, international, and academy soccer [6,7,8,9,10,11,12,13,14,15,16,17,29,30].

### 4.1. High-Speed and Sprint Demands

The high-speed demands of SD and W are compatible with their tactical responsibilities. Both roles commonly cover substantial longitudinal space during transitions, overlapping actions, and defensive recovery. In the adjusted models, CD completed approximately half the high-intensity running of W and fewer sprints than SD, ST, and W. This does not imply that central defending is physically undemanding; rather, its competitive profile is less dependent on repeated high-velocity running and more strongly shaped by tactical positioning and brief explosive actions. Similar positional contrasts have been reported in professional, international, and youth cohorts [6,7,8,9,10,11,12,13,14,15,16].

### 4.2. Acceleration and Deceleration Demands

Acceleration and deceleration outcomes provided information that was not captured by distance or peak speed alone. CD recorded fewer high-magnitude accelerations than the two wide groups and ST, and fewer decelerations than every other group. This pattern may reflect role-specific movement demands during match play. Central defenders typically operate within a more spatially constrained central area and rely more heavily on positional adjustments and short explosive actions, whereas wide defenders, wingers, and strikers are more frequently involved in pressing actions, transitions, recovery runs, and repeated changes in speed over larger spaces. These tactical demands may increase the frequency of rapid accelerations and braking actions in the latter positions [11,12,14]. The positional effect was particularly strong for braking actions. High-intensity decelerations involve substantial eccentric loading and may contribute to post-match neuromuscular stress even when total distance is moderate. Position-specific preparation should therefore include braking and re-acceleration demands, while acknowledging that event counts depend on device filtering and threshold definitions [11,12,14,27,29,31,32].

These positional differences should nevertheless be interpreted within the tactical structure of the team. The physical demands associated with the same nominal position may vary according to formation, pressing strategy, defensive organization, width, and specific in-possession and out-of-possession roles. Therefore, the broad positional categories used in the present study should be interpreted as team-specific role profiles rather than universal positional standards [13,14,30].

### 4.3. Exposure Adjustment and Threshold Interpretation

Exposure adjustment changed the interpretation of total distance. The unadjusted medians were heavily influenced by the longer match participation of CD and the shorter participation of ST. After actual duration and match were included in the model, CD accumulated less distance than M and W at equivalent exposure. Raw totals describe the dose actually accumulated and are relevant for recovery, whereas exposure-adjusted models address positional work rate while avoiding extreme extrapolation from short appearances. Previous research likewise shows that absolute and relative load indicators can produce different positional interpretations [10,18,19,20].

Peak speed should be interpreted as an interaction between tactical opportunity, match context, and individual capacity. M achieved a lower adjusted peak speed than SD, but a threshold such as 25 km/h does not represent the same relative intensity for every player. Individualized thresholds based on maximal sprint speed may alter the classification of high-speed running and sprint distance, particularly across positions with different speed capacities [5,21,22]. Future monitoring should ideally report both absolute and individualized high-speed indicators.

### 4.4. Practical Applications and Limitations

Practitioners should maintain position-specific reference profiles for total distance, high-intensity running, sprinting, peak speed, accelerations, and decelerations. Training for side defenders/full-backs and wingers should provide regular high-speed and sprint exposure, while all positions require braking and re-acceleration tasks matched to their competitive profile [12,15,18,19,27,31,32]. Short substitute appearances should not be interpreted through raw totals alone, but very brief exposures should also not be extrapolated uncritically to a full-match equivalent. These match-derived profiles complement training-based GPS evidence showing that pitch dimensions and player format materially alter internal and external load in semi-professional soccer [33].

Several limitations require emphasis. The sample represented one team, six matches, and 21 players, with three to five players per position. Position was defined using broad categories, and several contextual factors, including opponent strength, possession, and time-varying match state (e.g., score-line progression), were unavailable. One of the 85 individual source records lacked a position code and was excluded from the primary analysis. Absolute speed thresholds may misclassify relative intensity. Very short substitute appearances produced influential residuals, particularly for total distance; however, restricting the analysis to appearances lasting at least 30 min did not alter the conclusions. GPS accuracy is generally stronger for total distance and steady-speed running than for rapid changes in velocity, and manufacturer-specific processing may influence acceleration and deceleration outputs [4,25,26,27]. Although each player consistently wore the same 10 Hz Polar Team Pro unit in a standardized position on the upper back throughout the monitored period, firmware and software versions, proprietary filtering settings, satellite-signal quality metrics, and minimum event-duration criteria were not available in the retrospective export. These factors may affect between-study comparability [4,25,26,27,30].

The reported values should therefore be treated as team-specific reference data rather than universal normative standards. Replication across a larger number of teams and matches should use multilevel models that can separate player, match, position, and team effects. Incorporating individualized speed thresholds, tactical role, possession phase, and match context would further improve ecological interpretation.

## 5. Conclusions

Playing position was associated with professional soccer match-running and change-of-velocity demands after controlling for exposure time, match effects, and repeated observations from the same players. Side defenders/full-backs and wingers showed the most demanding high-speed profiles, while central defenders completed fewer sprints, accelerations, and decelerations at equivalent exposure. Position-specific monitoring should combine accumulated match dose with exposure-adjusted estimates and should not rely on total distance alone.

Overall, the findings largely supported the study hypotheses, particularly the expectation that wide positions would demonstrate greater high-speed demands and that accelerations and decelerations would show clear position-specific differences.

## Figures and Tables

**Table 1:** Descriptive match demands by playing position.

Variable	CD	SD	M	W	ST
Observations/players	14/4	15/4	22/5	22/5	11/3
Duration (min)	95.0 (94.2–99.1)	86.0 (74.2–95.0)	70.0 (23.0–87.5)	72.5 (29.5–88.0)	52.0 (16.0–78.5)
Total distance (m)	8620 (8198–8893)	9042 (7022–9821)	6409 (2668–9009)	7814 (3186–9633)	4951 (1844–8022)
HIR ≥19 km/h (m)	400 (314–467)	818 (547–1146)	444 (213–550)	770 (371–979)	331 (226–840)
Distance ≥25 km/h (m)	61 (24–86)	242 (114–294)	30 (17–87)	134 (101–258)	83 (61–1440)
Sprints ≥25 km/h (n)	4.0 (2.5–4.8)	13.0 (7.0–16.0)	3.5 (2.0–5.8)	8.5 (5.2–15.0)	5.0 (4.0–11.0)
Peak speed (km/h)	27.2 (26.7–29.9)	31.9 (30.2–32.7)	27.9 (26.6–28.7)	30.9 (30.1–32.9)	30.4 (27.8–31.2)
Accelerations ≥3 m/s^2^ (n)	11.5 (7.0–14.0)	18.0 (11.0–23.5)	11.0 (4.2–17.0)	17.0 (10.0–28.5)	12.0 (5.0–15.0)
Decelerations ≤−3 m/s^2^ (n)	19.5 (13.2–22.8)	28.0 (18.5–34.5)	21.5 (13.2–33.8)	33.5 (15.2–46.0)	15.0 (9.5–24.5)

Note: Values are median (interquartile range). CD, central defenders; M, midfielders; SD, side defenders/full-backs; ST, strikers; W, wingers; HIR, high-intensity running.

**Table 2:** Omnibus playing-position effects from linear mixed-effects models.

Outcome	Wald χ^2^ (df = 4)	Holm-Adjusted *p*
Total distance	20.69	0.002
HIR ≥19 km/h	13.29	0.020
Distance ≥25 km/h	11.70	0.020
Sprints ≥25 km/h	18.15	0.005
Peak speed	15.13	0.013
Accelerations ≥3 m/s^2^	20.70	0.002
Decelerations ≤−3 m/s^2^	53.36	<0.001

Note: Each model included playing position and match as fixed effects, centered log-transformed playing duration as a covariate, and player as a random intercept. Holm correction was applied across the seven prespecified outcomes.

**Table 3:** Significant Holm-adjusted pairwise positional contrasts.

Outcome	Pairwise Contrast	Adjusted Effect (95% CI)	Holm-Adjusted *p*
Total distance	CD vs. M	0.87 (0.81, 0.93)	<0.001
Total distance	CD vs. W	0.87 (0.81, 0.94)	0.001
HIR ≥19 km/h	CD vs. W	0.51 (0.34, 0.76)	0.010
Sprints ≥25 km/h	CD vs. SD	0.41 (0.24, 0.69)	0.007
Sprints ≥25 km/h	CD vs. ST	0.41 (0.23, 0.71)	0.015
Sprints ≥25 km/h	CD vs. W	0.45 (0.28, 0.75)	0.016
Peak speed	M vs. SD	−3.06 (−5.05, −1.07) km/h	0.026
Accelerations ≥3 m/s^2^	CD vs. SD	0.55 (0.39, 0.78)	0.006
Accelerations ≥3 m/s^2^	CD vs. ST	0.54 (0.37, 0.79)	0.012
Accelerations ≥3 m/s^2^	CD vs. W	0.50 (0.36, 0.69)	<0.001
Decelerations ≤−3 m/s^2^	CD vs. M	0.53 (0.42, 0.66)	<0.001
Decelerations ≤−3 m/s^2^	CD vs. SD	0.57 (0.45, 0.73)	<0.001
Decelerations ≤−3 m/s^2^	CD vs. ST	0.52 (0.40, 0.68)	<0.001
Decelerations ≤−3 m/s^2^	CD vs. W	0.44 (0.35, 0.55)	<0.001

Note: Ratios below 1.00 indicate lower values in the first-listed group. The peak-speed effect is an adjusted mean difference in km/h; all other effects are exponentiated ratios. CD, Central Defender; M, Midfielder; SD, Standard Deviation; ST, Striker; W, Winger.

## Data Availability

The anonymized data and analysis code supporting the findings of this study are available from the corresponding author upon reasonable request and subject to authorization by the club’s designated data controller. The data are not publicly available because of privacy and proprietary restrictions.
